# Asymmetric Bargaining Model for Water Resource Allocation over Transboundary Rivers

**DOI:** 10.3390/ijerph16101733

**Published:** 2019-05-16

**Authors:** Jianan Qin, Xiang Fu, Shaoming Peng, Yuni Xu, Jie Huang, Sha Huang

**Affiliations:** 1 State Key Laboratory of Water Resources and Hydropower Engineering Science, Wuhan University, Wuhan 430072, China; jnqin@whu.edu.cn (J.Q.); ynxu@whu.edu.cn (Y.X.); 2016102060058@whu.edu.cn (J.H.); huangsha247@163.com (S.H.); 2 Hubei Provincial Key Lab of Water System Science for Sponge City Construction, Wuhan University, Wuhan 430072, China; 3 Yellow River Engineering Consulting Co. Ltd., Zhengzhou 450003, China; pengshming@163.com

**Keywords:** transboundary water governance, water allocation, disagreement utility, asymmetrical negotiation power, asymmetric Nash bargaining solution (ANBS), Euphrates River Basin (ERB)

## Abstract

Sustainable transboundary water governance is often challenged by conflicts between agents, which necessitates the design of cooperative and self-enforcing alternatives to facilitate equitable water distribution. The Nash bargaining approach, which originated from game theory, could offer a good mathematical framework to simulate strategic interactions among involved agents by considering individual rational benefits. Given that river-sharing problems often involve multiple self-interested agents, the asymmetric Nash bargaining solution (ANBS) could be used to describe agents’ powers, as determined by disparate social, economic, and political as well as military status, and ensure win–win strategies based on individual rationality. This paper proposed an asymmetric bargaining model by combining multi-criteria decision making, bankruptcy theory, and the ANBS for water distribution in the transboundary river context. The Euphrates River Basin (ERB) with three littoral states was used as a case study. Turkey has the highest bargaining power in ERB negotiation since it dominates in terms of economic strength, political influence, and military capacity, whereas in the two downstream countries these aspects are limited due to their internal political fragmentation and weaker military status. The water satisfaction percentages of Turkey, Syria, and Iraq under the best alternative are 96.30%, 84.23%, and 40.88%, respectively. The findings highlight the necessity for synthetically considering the agent’s disagreement utility and asymmetrical power when negotiating over water allocation.

## 1. Introduction

Many parts of the world have long suffered from serious water shortage, intensified water conflicts, and deteriorating aquatic ecosystems amid dramatic human socioeconomic activities and significant climate changes [1,2]. As the chief source of renewable freshwater supply, flowing rivers have irreplaceable functions in natural ecology and social services because of their ability to adjust hydrological regimes, transport materials and energy, shape geology and geomorphology, and regulate surrounding climate changes. The water resources system in the transboundary river basin is usually associated with multiple local decision makers, who are generally self-optimizers, giving priority to their own objectives rather than broader state or national objectives [3]. The statistics from the Transboundary Freshwater Dispute Database show that approximately 276 transboundary rivers are shared by 148 riparian countries throughout the world [4], possess about 60% of the global river flows [5], and host more than 2.7 billion people [4,5]. Although many international water laws and conventions, including the Helsinki Rules [6], the UN Watercourses Convention [7], and the Berlin Rules [8], have been promulgated and implemented in an attempt to manage these rivers in an equitable and reasonable manner, they still failed to introduce internationally accepted and standardized allocation schemes for sharing these resources and their corresponding benefits [9,10,11], largely due to the absence of effective methods for synthetically considering the multidimensional attributes of river-sharing problems.

As a special natural resource, water resources have the attribute of quasi-public good in that in principle all stakeholders enjoy equal rights to access and use. Unilateral actions taken by self-optimizing decision makers may pose negative externalities for others as well as environmental systems, especially when water availability is not sufficient to cover claims. A situation which is often referred to as “the tragedy of the commons” will inevitably appear if the water resources system collapses due to water overuse. Nevertheless, it is probably excessively pessimistic to use this term to predict an inevitable tragedy for water resources [12], in the sense that resource users do not necessarily always follow the Nash strategies of the prisoner’s dilemma game with actions merely based on individual rationality [13]. Agents may self-organize in efforts to achieve a sustainable complex social-ecological system [14] through their learning process of improving adaptation and responsiveness to changing environmental and social conditions [15].

Conflict and cooperation coexist in the transboundary water governance [16,17,18], and the simultaneous consideration of these two processes helps to improve the quality of negotiation over water distributions [19,20]. Generally, three categories of institutions involving non-cooperation, cooperation, and exogenous regulation have been identified for managing common pool resources like water resources [21]. Non-cooperative institutions have been proven to not necessarily result in tragic outcomes [21], while a combination of laissez-faire and open access is very likely to trigger serious conflicts and even social instability. Effective exogenous regulation, for example in groundwater exploitation regulation, usually necessitates an authorized intervener (e.g., a social planner, government, or regulator), whose decisions are enforceable and acceptable by all agents, so this may be less practical in resolving realistic resource-sharing problems, particularly transboundary ones involving multiple local decision makers representing different or even conflicting interests. Cooperative institutions are obviously more conducive to the sustainable management of water resources and the long-term interests of agents involved [12].

Numerous theories and methods from various disciplines could be used to develop cooperative solutions for allocating internationally contested water resources among riparian agents. The social welfare maximization approach is often used to maximize the global gains with the assumption of a fully cooperative attitude among the agents involved, but it fails to distribute the total benefits among them, and often ignores their heterogeneity in terms of political, social, and economic status [22]; hence, the allocation solutions derived from this approach are not always practical [23]. Operations research-based allocation methods, such as compromise programming [24,25] and goal programming [26,27], usually focus on finding the social-optimal solutions by minimizing the sum of agent’s dissatisfaction with various solutions or the distance between the solutions and the ideal ones. Indeed, these distance-based methods [28] allow an increase in the system-level gains [29] compared with totally uncooperative mechanisms. Nevertheless, similar to the social welfare maximization approach, these methods distribute resources only from the social planner’s view with the assumption that perfect cooperation, transferable utility, and economic compensation exist among local agents [30], neglecting the interactions between them. Therefore, the practical feasibility of allocation solutions based on these methods is also likely questionable [28,31,32].

Analytical methods originated from the bankruptcy literature [33,34,35,36] have proved to be promising methods for addressing river bankruptcy problems [31]. As these distribution rules are normally based on common sense [10,31], concentrating on sharing resources among stakeholders directly according to their claims without the need to clarify their utility information and incremental benefits of cooperation, they have relatively good practical significance and flexibility in solving complex river-sharing problems, and the decision results are easily understood and accepted by local decision makers and policy makers [10,31,37]. However, these methods usually model the river bankruptcy problem as a cooperative transferable utility game [9] under the assumption that all agents involved have equivalent power in politics, society, and the economy, and in practice, these asymmetric factors inevitably influence each agent’s actions and decisions [28].

Water governance in the transboundary river context is complicated by overlapping interactions among the involved agents [38]. Correct understanding and dealing with these strategic interactions is critical to avoiding water conflicts [16] and achieving self-enforcing distribution agreements. Unpacking the discourse of water scarcity facilitates an understanding of agents’ causes, consequences, and interactions [39,40], greatly increasing the possibility of reaching a cooperation agreement. Solution concepts derived from cooperative game (CG) theory, such as the Nash bargaining solution, and the nuclear, the core and the Shapley value, have a strong potential to facilitate win–win solutions for sharing water resources and their benefits since they can well capture coalitional dynamics and multidimensional elements [23,41] affecting cooperation among the agents involved. Among these solution concepts, the Nash bargaining solution [42] has the ability to incorporate multiple desirable properties such as feasibility, invariance under changing utilities, Pareto optimality, and unanimity [43] into transboundary water sharing problems [9]. It has proved to be able to significantly balance the utilities among parties involved [44], and can serve as a base for sustainable river water-sharing agreements [45]. Hence, it is frequently used in the water resources literature [3,9,12,23,29,32,44,46,47,48,49,50,51,52,53,54] to address river-sharing issues involving multiple local decision makers that require consideration of the reliability criterion.

The transnational water management system could be described as a cooperative game with externalities [54]; effective negotiations among agents over water allocations, to a certain extent, can prevent hydro-hegemony [55]. However, the negotiation process is not necessarily based on the background of power symmetry [56], in the sense that agents likely have heterogeneous social, economic, political, and military positions that will inevitably influence the final negotiation outcome [57]. Asymmetry in power is a fundamental aspect of hydro-politics, which is one key element for conflict resolution in transboundary water governance [58,59]. Although asymmetrical powers between agents do not necessarily determine the results of negotiations, the overall benefits lie in the hands of the most powerful agent [60]. Therefore, the agents’ bargaining powers should be taken into account in river-sharing problems when available [28,55,56,58,59,60,61,62,63,64], as they help create a more realistic negotiation analysis [28]. The asymmetric Nash bargaining solution (ANBS) [65], generalized by the Nash bargaining solution [43], proved to be suitable for describing the agents’ asymmetrical negotiation powers through bargaining weights, and ensuring mutually beneficial resolutions for water-sharing problems. Nevertheless, it is both crucial and challenging to define agents’ bargaining weights (BWs) and disagreement utility points (DUPs).

Briefly, transboundary river management should be formulated as the multi-criteria decision-making (MCDM) problem bargained by multiple groups with disparate social, economic, political, and military status and needs [28,56,66]. The present study aims to develop an asymmetric bargaining model to find realistic and feasible solutions for river-sharing problems. The detailed operation steps are as follows: (1) data envelopment analysis (DEA) is selected as the MCDM approach to define agents’ negotiation powers (NPs) by synthetically considering their asymmetry in social, economic, political, and military aspects; (2) three methods based on the bankruptcy theory are developed to generate the minimum water rights (MWRs) for all participating agents with respect to their desired water needs and total available water resources, and then their DUPs are reckoned and considered as the starting points of bargaining in water allocation; (3) the ANBS is employed to establish an optimal allocation model for transboundary water resources by integrating the outcomes from the first two steps and some corresponding constraints; and (4) the proposed allocation framework is applied to the Euphrates River Basin (ERB), the center of one of the world’s insurmountable disputes involving the three sovereign littoral states of Turkey, Syria, and Iraq, for developing multiple allocation alternatives under different division scenarios, and four classical bankruptcy theoretical solutions are also derived for comparison.

The remainder of this paper is structured as follows. The next section will briefly describe the case study of the ERB, and then systematically present the proposed allocation framework and examine relevant methods. Section 3 will report possible allocation results and corresponding discussion. Finally, the conclusions will be summarized in Section 4.

## 2. Materials and Methods

### 2.1. Study Area

**Figure 1:**
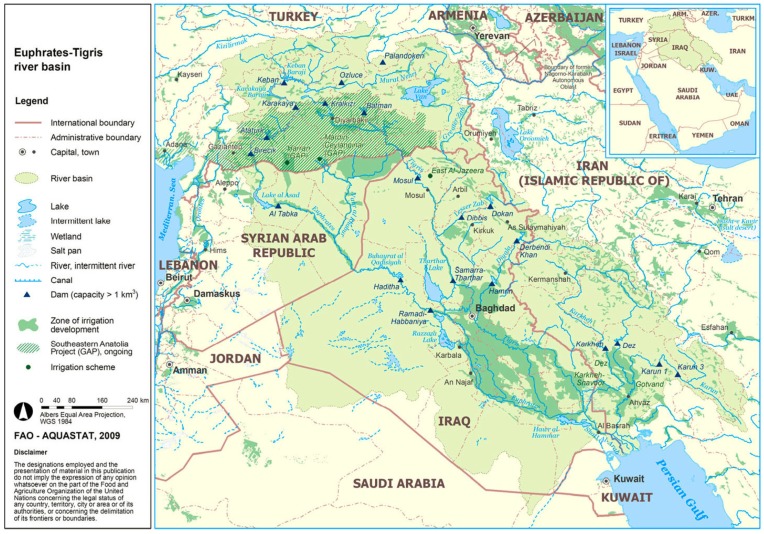
Euphrates—Tigris River Basin. (FAO-AQUASTAT: AQUASTAT Main Database, Food and Agriculture Organization of the United Nations 2009) [67].

The Euphrates and Tigris Rivers (the Two Rivers) originate in the Anatolian Highlands of Turkey, flow through Syria and Iraq, and finally join the Persian Gulf (Figure 1) [67], forming a major river system in Western Asia with its tributaries. According to the research reports of some scholars on irrigable land and water demand as well as hydro-policies of each riparian country for the Two Rivers [10,68,69,70,71,72], the total length of Euphrates River is about 3000 km, with Turkey in the upstream accounting for 41%, Syria in the middle reaches accounting for 23%, and Iraq accounting for 36% (Table 1). Turkey contributes the most of 31,580 million cubic meters per year (MCM/year) to the Euphrates River runoff, approximately eight times more than Syria (4000 MCM/year), whereas Iraq makes no contribution. The total available water resources within the ERB are 35,580 MCM/year, but the total water claim is 54,700 MCM/year. Therefore, the water resources in this basin are not sufficient to feed its three riparian countries, and competition is inevitable in the allocation of water rights.

Upstream Turkey has the opportunity and ability to control a large proportion of the river flow for thoroughly serving its industry and agriculture, and even exporting water to other countries, since most of the river’s tributaries are located in this country. In contrast, the two downstream countries of Iraq and Syria, which depend mainly on the river for drinking, industrial and agricultural development, and power generation, frequently suffer from water shortages. Prior to 1960, hydropolitical relations regarding the Two Rivers among the three littoral states could be characterized as harmonious [73]. Since then, their relations have become tense, especially after Turkey implemented the Southeastern Anatolia Project or Guneydogu Anadolu Projesi (GAP) aimed at harvesting water for irrigation, hydroelectric energy, and addressing its Kurdish ethno-political concerns through the building of 22 dams and 19 hydropower plants on the Two Rivers [59,60,74]. According to international experts, a full implementation of the GAP will ultimately withdraw a maximum of 70% and 50% of the natural runoff of the Euphrates and Tigris Rivers, respectively [60].

Normally, the unilateral construction of hydropower facilities on transnational rivers is likely to be unsuccessful [64,74] unless it is aligned with the geopolitical interests of the riparian states [75]. However, the GAP project of Turkey has been successfully implemented [74] despite the fact that was once considered as a significant threat and was strongly resisted by the downstream states [73]. Apart from its absolute geographical and hydrological advantages, Turkey dominates in terms of technical expertise, economic strength, regional and global political influence, and military capacity [74], which gives it a relatively high influential capacity in the negotiation over water allocations, or even unilateral actions regardless of the multilateral agreements already signed [76]. In terms of the demographic and economic aspects, Turkey has the largest population (67.90 million in 2005), 2.51 and 3.71 times than that of Iraq (27.01 million in 2005) and Syria (18.29 million in 2005), respectively. The economic status of the three states exhibits even more disparity; the gross domestic product (GDP) of the Turkey was $50,141.63 million in 2005, which is, respectively, 10.03 and 17.37 times that of Iraq ($4995.49 million) and Syria ($2885.90 million) in the same year. Therefore, Turkey is more capable of influencing the outcome of Euphrates River negotiations, or is more likely to violate agreements already signed when water shortages occur just from the perspective of population and economic development. Nevertheless, it is also worth noting that water resources are common pool resources that all stakeholders enjoy equal rights to access and use. Moreover, the two downstream countries are highly dependent on the Euphrates River for developing their society and economy; they may risk armed conflicts for water resources if upstream Turkey overutilizes water resources under situations of water extreme shortage.

As analyzed above, power asymmetries among three littoral states inevitably affect Euphrates River negotiations [60], which are determined by multidimensional nature of society, politics, economy, and military affairs [28,56,57]. For estimating agents’ bargaining powers in the ERB, this study identifies some criteria and associated indicators referring to the indicator system for determining the five riparian countries’ capabilities in the Caspian Sea negotiations constructed by Sheikhmohammady et al. [56]. Table 2 lists all the criteria considered as important determinants of the countries’ capabilities in the ERB. The criteria for economic independence and self-sufficiency include the gross national income (GNI)/capita, Gini coefficient, net trade/GDP, and GDP/energy consumption. Military status includes yearly military expenditure, military expenditure/GDP, and armed forces personnel/total population. International support involves U.S. financial, U.S. political, and Russian political support. Political influence and structure consist of political influence and democracy level. The specific significance and determination methods of various indicators in each criterion are discussed in detail in the literature of Sheikhmohammady et al. [56]. It is noteworthy that although the indicator system suggested by Sheikhmohammady et al. [56] is used for reference, the selection of some indicators is adjusted in the current work because different basins exhibit different attributes. Relevant statistics are mainly extracted from the World Bank [77], the U.S. Aid Budget [78], the Economist Intelligence Unit [79], and related research literature [10,68,69,70,71,72].

### 2.2. Methods

In this section, the suggested asymmetric bargaining framework for transboundary river-sharing problems will be depicted in detail, and are constituted by four main parts, namely, general river bankruptcy problems, the ANBS, asymmetric agents’ NPs, and agents’ DUPs.

#### 2.2.1. River Bankruptcy Problems

A river bankruptcy problem is a particular case of distribution problem in which limited water resources must be distributed but are not sufficient to cover all the agents’ claims. This problem is usually formulated as a triple (N,E,c) in accordance with its definition above [10]; here, we consider: (1) a finite set of agents N=[1,2,…,n] (usually administrative regions or riparian countries within a transboundary river basin) who are generally self-optimizers competing for limited water resources according to their own objectives; (2) limited available water resources E, which should be divided among these agents; and (3) each agent’s aspiration claim $c_{i}$, and i∈N. Suppose that $a_{i}$ is the available water (or water contribution) in the territory of riparian i; thus the total available water resources within the river basin are $E=\sum_{i=1}^{n}a_{i}$. Competitive conflicts will arise among involved agents, and existing distribution agreements may fail when the sum of their claims exceeds the distributed water asset, that is, $C=\sum_{i=1}^{n}c_{i}>E$. At this time, optimization techniques are needed to reasonably allocate the water resources for buffering conflicts and preventing the collapse of the water resource system. Assuming that $x_{i}$ is the water resource allocated to agent i, three preconditions of bankruptcy distribution problems must be met: (1) Pareto efficiency, $\sum_{i=1}^{n}x_{i}=E$, which requires all aggregate values (available water resources) of the grand coalition to be exactly apportioned among individual agents; (2) claim boundedness, $x_{i}\leq c_{i}$, which helps prevent resource overuse that may cause the tragedy of the commons; and (3) non-negativity, $x_{i}\geq 0$ [80].

#### 2.2.2. Asymmetric Nash Bargaining Solution

The Nash bargaining solution has a strong potential to find win–win solutions for sharing water resources and their benefits since they can capture well coalitional dynamics [23,41], and has the ability to take most of the desirable properties of the river-sharing problem into account [9]. As river-sharing problems in reality have asymmetric attributes, the ANBS [65] has attracted considerable attention from water resource scientists, and has achieved remarkable results in some river conflicts including the Mekong River Basin [52], the Mexican Valley [53], the Huai River Basin [50], and the Aharchay River Basin [47]. Nevertheless, most of these studies make excessive assumptions about actual examples, especially in determining BWs. Other studies directly use hypothetical examples as research objects [9,51,57], and few provide allocation solutions for conflict resolution in practical cases, such as that of the Euphrates River.

In the asymmetric Nash bargaining theory, players’ preferences (presented by utility functions), their disagreement points, and individual risk-taking attitudes are explicitly considered [81]. Suppose that a river bankruptcy problem is an *n*-player game, and Ω is the decision space that contains a set of potential utility profiles. $u_{i}\left(x_{i}\right)\in \Omega$ is the utility function of player i. $u_{i}^{0}\left(m_{i}\right)$ is defined as the utility value of player i at its disagreement point. $\omega_{i}$ is the BWs of player i, and $\sum_{i=1}^{n}\omega_{i}=1$. A unique solution can be obtained if the utility function space is proven to be convex, closed, and bounded [42,65]. An asymmetric bargaining model for river water allocation that considers multidimensional influence factors can be established by integrating the agents’ BWs, heterogeneous DUPs, the ANBS, and corresponding constraints. The mathematical expression of the model is given as follows:

(1) $$Z=\max{\prod_{i=1}^{n}\left[u_{i}\left(x_{i}\right)-u_{i}^{0}\left(m_{i}\right)\right]}^{\omega_{i}}$$

subject to

(2) $$u_{i}\left(x_{i}\right)\geq u_{i}^{0}\left(m_{i}\right)$$

where Z is the objective function of the *n*-player bargaining game which ensures that the game has the ultimate unique solution of the game. Equation (2) denotes the individual rationality condition that requires the utility of each player after bargaining to be greater than its DUP [23]; otherwise, this player may break away from cooperative alliances. Assigning different values to parameter $\omega_{i}$ enables it to explore various possible alternatives under different influencing factors for identifying flexible, self-enforcing, and sustainable solutions. For $\omega_{i}=1$, this denotes that all the agents are given equal BW values of 1, and that the river-sharing problems are a type of symmetric game problem. For $\omega_{i}=\omega^{Rc}$, this represents that the BW vector of the agents is determined only by their water claim ratios. For $\omega_{i}=\omega^{Ra}$, this indicates that the BW vector of the agents is determined only by their water contribution ratios. For $\omega_{i}=\omega^{NP}$, this indicates that the BW vector of the agents is determined by their NPs. From Equation (1) it can intuitively be seen that agents’ BWs and their heterogeneous DUPs are crucial in obtaining the value of Z and its corresponding Nash bargaining solution. In addition, the asymmetric bargaining model for river water allocation must obey the three constraints in bankruptcy distribution problems, namely, Pareto efficiency, claim boundedness, and non-negativity.

The linear ratio of agents’ water allocations to their claims can directly reflect their satisfaction degree with a given solution [28,82], and can thus be used to denote their utility functions [83] in the asymmetric bargaining water allocation model. Therefore, on the basis of water allocations (decision variable), MWRs (decision variable), and the aspiration water claims (known variable) of the agents, their allocation utility value and DUPs can be calculated by using the following proposed mathematical formulations, respectively:

(3) $$u_{i}\left(x_{i}\right)=x_{i}/c_{i}$$

(4) $$u_{i}^{0}\left(m_{i}\right)=m_{i}/c_{i}$$

subject to

(5) $$0\leq m_{i}\leq c_{i}$$

where $m_{i}$ is the MWR of agent i. The other abbreviations are mentioned above.

#### 2.2.3. Agents’ Negotiation Power

In physics, power has a precise definition, defined simply as work done divided by the time taken to accomplish it, whereas in political science it is vague [56]. A widely accepted political definition of power is the ability of one party to move another party in an intended direction, which is often related to GDP, population sizes, political influence, and military strength. In transboundary water governance, agents’ asymmetric powers may prevent cooperation [57] and affect the final negotiation outcomes, so they must be estimated quantitatively [56], and taken into account in river-sharing problems when available [28,55,56,58,59,60,61,62,63,64]. Mianabadi et al. [71] determined heterogeneous negotiation (power) weights for the three countries of the Euphrates River based on the environmental crisis within their territory. On the basis of considering exogenous factors including economic independence and self-sufficiency, military status, financial and military support from superpowers, and political influence and structure, Sheikhmohammady et al. [56] selected DEA as the MCDM approach to determine the five littoral states’ legal status in the Caspian Sea negotiations. According to the Article 6 of the UN Watercourses Convention [7], Zeng et al. [84] developed an indicator system on equitable and reasonable water allocation for the Guanting River Basin in China, and applied projection pursuit as the MCDM approach to synthesize values of these indicators to defined negotiator weights for 10 administrative regions.

Drawing on the indicator system constructed by Sheikhmohammady et al. [56] for the Caspian Sea negotiation, the present study lists all the criteria related to the legal status of three sovereign riparian states in the ERB and identifies the associated indicators for each criterion (Table 2). Once the indicator system is established, appropriate methods should be selected to integrate these indicators into comprehensive weight values for each agent. Several methods may be available for determining the priority or weight vectors in addressing multi-criteria evaluation problems, such as the principal component analysis [85], the system dynamics model [86], the technique for order preference by similarity to an ideal solution (TOPSIS) [87], the projection pursuit [84], the artificial neural network model [88], the clustering analysis [89], the analytic hierarchy process [90], and the DEA [28,56]. Each method has its advantages and corresponding applicable conditions. DEA, originally created by Charnes et al. [91], is a nonparametric efficiency evaluation method based on relative efficiency theory. This method firstly treats each evaluation unit as a decision making unit (DMU), and takes the index weights of each DMU’s inputs and outputs as variables; subsequently, it applies convex analysis and linear programming as tools to calculate the effective frontier of production. Ultimately, it measures the relative efficiency of each DMU with respect to the relative distances between it and the effective frontier [91].

According to the inherent principle of the aforementioned method, its advantages can be summarized as follows: (1) it can easily be used and has an absolute advantage in dealing with multiple input and output effectiveness evaluation problems; (2) the optimal efficiency index of the DMU is not related to the dimension selection of the input and output index values, and it is not necessary to dimension the data before using the method to build the model due to it not directly integrating the statistical data; (3) this method does not require any weighting assumptions, and the optimal weight vector is obtained by the actual data of DMU’s inputs and outputs, so it can eliminate many subjective factors and exhibits strong objectivity; and (4) it assumes that each input is associated with one or more outputs, and highlights some connection between inputs and output, but it does not require the explicit expression of this relationship to be determined. Due to the inherent advantages mentioned above, DEA has achieved remarkable results in various evaluation problems with multiple variables in recent years [28,56,92]. Hence, this paper selects DEA to determine agents’ NPs affected by multiple factors in river-sharing problems, and the fundamental model is proposed as follows:

(I) Related variables

We consider a set of DMUs K=[1,2,…,k] in a multi-criteria evaluation problem, where h∈K and each DMU has L=[1,2,…,l] types of inputs and S=[1,2,…,s] types of outputs, with p∈L and r∈S. Then, the input and output vectors of DMU h are $x_{h}={\left(x_{1h},x_{2h},\ldots ,x_{lh}\right)}^{T}$ and $y_{h}={\left(y_{1h},y_{2h},\ldots ,y_{sh}\right)}^{T}$, respectively, where $x_{ph}$ is the input amount of the DMU h to the input type p, and $x_{rh}$ is the output amount of the DMU h to the output type r.

(II) Efficiency evaluation index of DMU

In order to “integrate” all inputs and outputs, that is, to treat them as a production process with only one overall input and output, each of them must be given the correct weight. Assuming that the weight vectors of the inputs and outputs are $\nu ={\left(\nu_{1},\nu_{2},\ldots ,\nu_{m}\right)}^{T}$ and $\mu ={\left(\mu_{1},\mu_{2},\ldots ,\mu_{s}\right)}^{T}$, the efficiency evaluation index of DMU h can be expressed as follows:

(6) $$\theta_{h}=\sum_{r=1}^{s}\left(\mu_{r}\cdot y_{rh}\right)/\sum_{p=1}^{l}\left(\nu_{p}\cdot x_{ph}\right)$$

subject to

(7) $$\nu ={\left(\nu_{1},\nu_{2},\ldots ,\nu_{m}\right)}^{T}\geq 0\;\forall m$$

(8) $$\mu ={\left(\mu_{1},\mu_{2},\ldots ,\mu_{s}\right)}^{T}\geq 0\;\forall s$$

where $\theta_{h}$ is the efficiency evaluation index value of the DMU h, $\nu_{p}$ is the weight value of the input type p, $\mu_{r}$ is the weight value of the output type r, $\sum_{p=1}^{l}\left(\nu_{p}\cdot x_{ph}\right)$ is the input comprehensive value of the DMU h, and $\sum_{r=1}^{s}\left(\mu_{r}\cdot y_{rh}\right)$ is the output comprehensive value of the DMU h. The other abbreviations are mentioned above.

(III) Relative efficiency optimization evaluation model

$x_{ph}$ and $y_{rh}$ are known variables obtained from historical statistics or predictive data. Thus, the actual problem is to determine a set of optimal weight vectors (ν and μ) for maximizing the efficiency evaluation index value of the DMU h ($\theta_{h}$), which is a relative efficiency evaluation value of the DMU that cannot possibly be higher than those of the other DMUs.

The relative efficiency optimization evaluation model of the DMU i, i∈K, can be proposed as follows:

(9) $$\max\theta_{i}=\sum_{r=1}^{s}\left(\mu_{r}\cdot y_{ri}\right)/\sum_{p=1}^{l}\left(\nu_{p}\cdot x_{pi}\right)$$

subject to

(10) $$\theta_{i}<1,\;\forall i$$

where $\theta_{i}$ is the efficiency evaluation index value of DMU i, $\sum_{p=1}^{l}\left(\nu_{p}\cdot x_{pi}\right)$ is the input comprehensive value of the DMU i, and $\sum_{r=1}^{s}\left(\mu_{r}\cdot y_{ri}\right)$ is the output comprehensive value of the DMU i. The other abbreviations are mentioned above.

(IV) Linear transformation of the model

The relative efficiency optimization evaluation model established above is a fractional programming model, which is inconvenient to use directly in multi-criteria evaluation problems. Therefore, this paper uses the Charnes–Cooper transformation technique [93] to convert it into a linear programming model. Let $t=1/\sum_{p=1}^{l}\left(\nu_{p}\cdot x_{pi}\right)$, $\eta_{r}=t\cdot \mu_{r}$ and $w_{p}=t\cdot \nu_{p}$, then, the original fractional programming model can be transformed into:

(11) $$\max\beta_{i}=\sum_{r=1}^{s}\left(\eta_{r}\cdot y_{ri}\right)$$

subject to

(12) $$\sum_{r=1}^{s}\left(\eta_{r}\cdot y_{rh}\right)-\sum_{p=1}^{l}\left(w_{p}\cdot x_{ph}\right)\leq 0,\;\forall h,r,p$$

(13) $$\sum_{p=1}^{l}\left(w_{p}\cdot x_{pi}\right)=1,\;\forall i,p$$

(14) $$\eta_{r}\geq 0,w_{p}\geq 0,\;\forall r,p$$

(V) Standards for efficiency evaluation of DMU

The linear transformation model runs k times, and all DMUs can receive their optimal efficiency value. The relative efficiency can be calculated by the following formula:

(15) $$RE_{i}=\beta_{i}/\sum_{i=1}^{k}\beta_{i}$$

The relative efficiency values of all DMUs are less than or equal to 1. If $RE_{i}=1$, then DMU i is the most productive among all DMUs or is relatively effective for the system. If $RE_{i}<1$, then the productivity of the DMU i should still be improved relative to the other DMUs, or it is not yet effective for the system.

(VI) Agents’ NPs

Influencing factors from political, social and economic perspectives are aggregated into the NPs (relative efficiency values) by DEA in river-sharing problems, and which are described as follows:

(16) $$\omega_{i}^{NP}=RE_{i}$$

subject to

(17) $$\sum_{i=1}^{n}\omega_{i}^{NP}=1$$

(18) $$0\leq \omega_{i}^{NP}\leq 1$$

where $\omega_{i}^{NP}$ is the NP of agent i, $RE_{i}$ is the relative efficiency values of DMU (agent) i, and i∈N=K. The other abbreviations are mentioned above.

#### 2.2.4. Agent’s Disagreement Utility Point

In the Nash bargaining theory, the individual rationality condition (Equation 2) is the core and basis of forming a negotiating alliance [23]. Agent’s disagreement utility is the lower bound or starting point for his participation in bargaining [54], which, to some extent, determines the fairness and sustainability of existing treaties or agreements [51]. Therefore, the ANBS depends upon agents’ DUPs [47,51]. The agents’ gains in status quo are always directly used as their DUPs, but such gains may not exist in actual bankruptcy problems [31], and likely result in erroneous results [53] even if they do exist. Therefore, effective alternative methods are needed to define agents’ MWRs for supporting the smooth progress of negotiations. Some studies suggest that the bankruptcy theory method can be used to determine the MWRs for agents involved, and then determine their DUPs [9,31,50]. Furthermore, given the fact that the traditional bankruptcy rule may not be able to allocate certain initial water rights to the agents with low claims when resources are in extreme shortage, this paper proposes two additional methods to define the MWRs for all agents involved based on the proportional (PRO) rule.

##### Method Based on Traditional Bankruptcy Theory (MTBT)

The mathematical formulation based on the traditional bankruptcy theory [33] for determining agents’ MWRs is proposed as follows:

(19) $$m_{i}=\left\{0,E-\sum_{j\in N-\left\{i\right\}}c_{j}\right\}$$

subject to

(20) $$0\leq m_{i}\leq c_{i}$$

(21) $$E\leq \sum_{i=1}^{n}c_{i}$$

where $m_{i}$ is the MWR of agent i produced by traditional bankruptcy theory, which is the amount of water conceded to agent i by all other agents [31,33]. The rationale of traditional bankruptcy theory is that each agent receives a minimum initial water right equal to the water amount that remains if all the other agents have been fully compensated in terms of their target claims [90]. $\sum_{j\in N-\left\{i\right\}}c_{j}$ is the sum of the water claims of the agents except i, $c_{i}$ and $c_{j}$ are the aspiration water claims of agent i and j, respectively, and i≠j∈N.

##### Two other methods based on PRO

The PRO, probably the best known and most widely used solution in the literature on bankruptcy problems [10,35], is defined by making the awards proportional to the aspiration claims truncated by the amount for division [36], and this proportionality is often taken as the definition of fairness for claims problems [36,94]. The PRO can be defined as follows:

(22) $$PRO(x_{i})=\lambda_{PRO}\cdot c_{i}$$

(23) $$\lambda_{PRO}=\sum_{i=1}^{n}a_{i}/\sum_{i=1}^{n}c_{i}$$

where $\lambda_{PRO}$ is the fixed proportional coefficient of each agent with respect to their aspiration claims, and is chosen for Pareto efficiency [36].

It may be considered equitable and reasonable that agents who contribute more and claim less bear a smaller share of water shortage [10] or should be granted a higher priority while allocating water resources. Based on this viewpoint and the PRO division, the first method for defining agents’ MWRs is proposed with respect to their rates of demand and contribution (MRDC), and is defined as:

(24) $$m_{i}=\alpha \cdot PRO\left(x_{i}\right)$$

(25) $$\alpha =\left(Ra_{i}+1-Rc_{i}\right)/n$$

(26) $$Ra_{i}=a_{i}/\sum_{i=1}^{n}a_{i}$$

(27) $$Rc_{i}=c_{i}/\sum_{i=1}^{n}c_{i}$$

where α is proportional coefficient of the PRO distribution for agent i based on its rates of claim and contribution, $Ra_{i}$ is the water contribution rate of agent i, and $Rc_{i}$ is the water claim rate of agent i.

Another method based on the PRO division for defining agents’ MWRs is supposed with respect to their external NP (MENP), and is defined as follows:

(28) $$m_{i}=\omega_{i}^{NP}\cdot PRO\left(x_{i}\right)$$

Here, all abbreviations are the same as those defined previously.

## 3. Results and Discussion

### 3.1. NPs of Three Countries in the ERB

Agents who dominate in terms of technical expertise, economic strength, regional and global political influence, and military capacity might have a relatively high influential capacity in negotiations over water allocations [74]. Hence, it is necessary to ensure that the asymmetric information is synchronously integrated into the derivation of distribution alternatives. On the basis of the defined indicators shown in Table 2, the DEA method is used to derive the maximum efficiency value of each agent (out of 10) in the ERB by using the linear programming method to find a set of weight vectors that can maximize the efficiency of each agent. Iterative calculation is carried out three times, the optimal efficiency values of the three riparian countries in the ERB are obtained, and the NPs of three countries are defined by standardizing their optimal efficiency values (Table 3).

From Table 3, Turkey has the highest optimal efficiency value of 8.7485 as it dominates the two other countries in seven out of 12 evaluation indicators, which enables it to have the most NP in the Euphrates River negotiations. In terms of economic independence and self-sufficiency, military status, as well as political influence and structure, Iraq is obviously the weakest among the three countries; hence, its optimal efficiency value is the lowest at 5.8474. As most of the evaluation indicators values are intermediate, Syria receives the median optimal efficiency value of 7.0802. Agents’ NPs can be calculated by standardizing their optimal efficiency values. As anticipated, Turkey is identified as the most powerful country, followed by Syria and Iraq. These NPs can be used as agents’ BWs in the asymmetric Nash allocation model for creating a more realistic negotiation analysis [28].

### 3.2. DUPs for the Three Countries in the ERB

The agent’s DUP is another necessary condition for bargaining model for water allocation in the ERB. To this end, three methods based on bankruptcy theory are developed to produce the MWRs of the three countries in the ERB, and their DUPs are subsequently derived by using Equation (4). Table 4 shows the MWRs and DUPs for the three riparian countries in the studying basin under the three methods. Iraq receives an initial water right of 8980 MCM/year under the MTBT, whereas Turkey and Syria get zero. The main reason is that the surplus water amount that can be used as the initial water allocation to Iraq under the MTBT after Turkey and Syria is 100%. The water demand of Iraq is the largest, reaching 51.38% of the total water demand, whereas the sum of the water demands of the two other countries is only 48.62%, which is significantly less than the total available resources (100%). Therefore, a certain water amount can be preferentially allocated to Iraq under the premise that the remaining resources can fully meet the needs of the other countries [33]. In contrast to the results of the MTBT, the other two methods based on the PRO allocation lead to the initial water rights for all three agents. MRDC gives the most initial water to Turkey (4952.78 MCM/year), whereas MENP allocates the most initial water (4930.70 MCM/year) to Iraq. Although parameter α and the NP values of Iraq both are the lowest under the two methods, the country receives relatively high initial water rights as the PRO allocates the most shares to the agent with a relatively high claim [33]. Syria obtains the minimum initial water rights under the two methods. From the numerical results in Table 4, it can be affirmed that Iraq is more likely than the other countries to be reluctant to participate in cooperative grand alliances if its DUP is not pre-satisfied, since it highly depends on the water resources of Euphrates River for residential domesticity, industry, agriculture, and energy purposes, and claims the most among all three countries in the ERB.

### 3.3. Solutions under Different Division Scenarios

**Figure 2:**
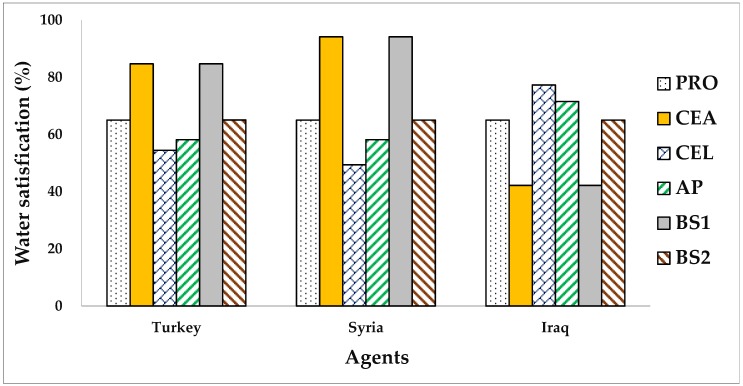
Water satisfactions (%) of three agents under four classical bankruptcy solutions and two Nash bargaining solutions. BS1: bargaining solution under d_i_ = 0 and ω_i_ = 1; BS2: bargaining solution under d_i_ = 0 and ω_i_ = ω^Rc.^; PRO: proportional; CEA: constrained equal award; CEL: constrained equal loss; AP: adjusted proportional.

**Figure 3:**
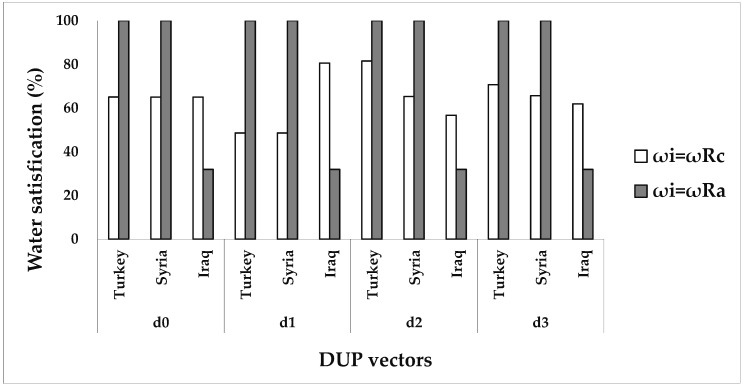
Water satisfactions (%) of three agents under two Nash bargaining solutions with considering different DUP vectors and two bargaining weights of ω^R^^c^ and ω^Ra^. d0 indicates that the agents’ DUP vector is 0; d1 indicates that the agents’ DUP vector is determined by MTBT; d2 indicates that the agents’ DUP vector is determined by MRDC; d3 indicates that the agents’ DUP vector is determined by MENP.

**Figure 4:**
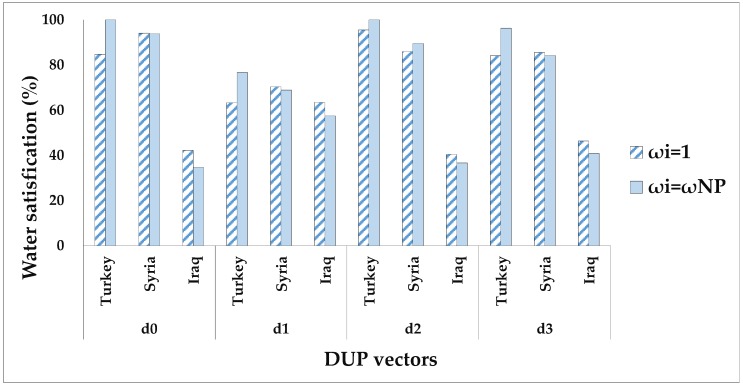
Water satisfactions (%) of three agents under two Nash bargaining solutions considering different DUP vectors and two bargaining weights of 1 and ω^NP^. d0: indicates that the agents’ DUP vector is 0; d1 indicates that the agents’ DUP vector is determined by MTBT; d2 indicates that the agents’ DUP vector is determined by MRDC; d3 indicates that the agents’ DUP vector is determined by MENP.

**Figure 5:**
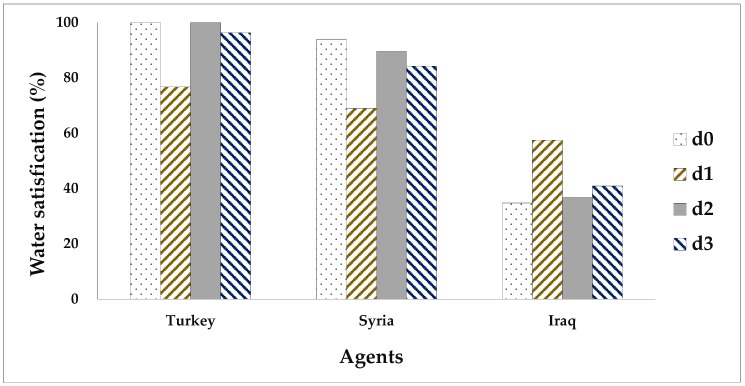
Water satisfaction (%) of three agents under four Nash bargaining solutions considering different DUP vectors and ω^NP^. d0 indicates that the agents’ DUP vector is 0; d1 indicates that the agents’ DUP vector is determined by MTBT; d2 indicates that the agents’ DUP vector is determined by MRDC; d3 indicates that the agents’ DUP vector is determined by MENP.

The suggested allocation framework is run under different division scenarios in view of whether or not to consider agents’ DUPs and their NPs. Four classical bankruptcy rules, namely, the PRO, the constrained equal award (CEA), constrained equal loss (CEL), and adjusted proportional (AP), are used for comparison purposes. Table 5 and Table 6 report the allocation solutions derived from the different methods. For facilitating the comparative analysis among different solutions, this paper converts the water allocations obtained by the agents under various solutions into the form of satisfaction of their claims, that is, the ratio of water amount allocated by each agent to their claims. The results are shown in Figure 2, Figure 3, Figure 4 and Figure 5.

In Figure 2, the CEA attempts to satisfy those agents with low claims as much as possible to reduce the number of dissatisfied ones [35], and thus, it favors agents with lower claims [9,31,33,35]. For example, this rule satisfies the water claim of Syria with 94.13% since it has the lowest claim, while for Iraq it only satisfies 42.21%. It is proven as the unique Lorenz maximizing rationing rule among the classical bankruptcy rules since no other rule results in more equally distributed shares [95]. Contrary to the CEA, the CEL focuses on minimizing the gap between claim and deficit [35], and all agents will share the same amount of loss. This rule gives priority to satisfying agents with the highest claim first, and once it is satisfied, the process is repeated with the remaining resources and agents [31]. Notably, the CEL probably does not give resources to the agent with the lowest claim when resources are extremely scarce. Turkey, Syria, and Iraq bear the same water shortage of 6373.33 MCM/year under the CEL solution, whereas their water satisfaction levels, respectively, are 54.48%, 49.42%, and 77.32%. Syria is severely penalized by CEL since its lowest water claim. This rule considered to be the unique Lorenz minimizing rationing rule among the classical bankruptcy rules due to no other rule leads to less equally distributed shares [35].

The PRO allocates to each agent a same proportional to their claims, thus, the three littoral states in the ERB receive the same water satisfaction of 65.05%. Although this rule treats every claim equally, it will give a relatively higher resource share to parities with high claims. Iraq is allocated the highest water share of 51.37% by this rule as it claims water the most, approximately 2.23 times the claim of Syria. Obviously, this rule is biased toward higher claims in resource allocations [31]. The AP gives each agent an initial water rights, and then uses the PRO to distribute the remainder with respect to the agent’s adjusted claims [33,36]. By comparing four classical bankruptcy solutions in Figure 2, the PRO and AP can be identified as moderate allocation methods even if they allocate the relatively high water share to parties with higher claims. The four alternative bankruptcy rules allocate the water resources of the Euphrates River to its three littoral states only with respect to their claim values; neither their contributions to the total available resources nor asymmetric social, economic, and political status are incorporated into the allocation process. Therefore, the realistic feasibility of these allocation solutions is questioned.

The allocation solution derived from the proposed allocation framework is actually a symmetric Nash bargaining solution when the agents involved are given equal BWs. To some extent, the symmetric Nash bargaining solution can be regarded as a special case of asymmetric Nash bargaining solution [9]; that is, the symmetric problems can be transformed into an asymmetric one by assigning different BWs to the involved players. By comparing four classical bankruptcy solutions with two Nash bargaining solutions (BS1 and BS2) in Figure 2, it can be found that the allocation solution produced by the proposed allocation framework (BS1) is the same as that obtained by the CEA when agents’ DUPs are not considered (d_i_ = 0) and their BWs are equal (ω_i_ = 1), which verifies the research conclusions drawn by Degefu et al. [9] and Dagan and Volij [34] to some extent. Analogously, the allocation solution generated by the proposed allocation framework (BS2) is almost identical to the PRO solution when agents’ DUPs are not considered (d_i_ = 0) and their BWs are derived from their claims (ω_i_ = ω^Rc^), which was also verified by Degefu et al. [9] and Dagan and Volij [34].

However, different results are generated when agents’ DUPs and their asymmetric BWs are considered. In Figure 3, the asymmetric Nash bargaining solutions under four different DUP vectors are the same when the asymmetric BWs for the three riparian countries are determined only by their water contribution (ω_i_ = ω^Ra^). For instance, Turkey and Syria are satisfied 100% under the four allocation solutions above, whereas Iraq receives the remaining water, and is only 31.96% satisfied. It can be seen that distributing water rights in the ERB solely on the basis of contributions is not advisable, in the sense that Iraq becomes highly dissatisfied and then resolutely resists implementing these solutions. Relative to four solutions under ω^Ra^, Iraq receives more water satisfaction under four solutions under ω^Rc^ despite its satisfaction is different under four DUP vectors, whereas the water satisfactions of the other two countries are both reduced. Taking the DUPs produced by MENP as an example, the water satisfactions of Turkey and Syria under ω^Rc^, are reduced by 29.32% and 34.34%, respectively, in comparison with those under ω^Ra^, while that of Iraq is increased by 30.01% because of its highest claim. Iraq receives the lowest satisfaction level relative to the other two countries, but this country actually is given the highest resource share, and thus, two other countries, especially Turkey, would oppose the solutions under ω^Rc^.

In Figure 4, the four solutions under equal BWs (ω_i_ = 1) seem to be fair for the three countries in the ERB; nevertheless, the disparate social, economic, and political status of the three countries certainly affects the negotiations over water allocations, making these solutions less practical. Finally, the four solutions under asymmetric NPs should be relatively more realistic since these BWs are calculated for each party based on their political, economic, and social status. For example, relative to the four symmetric solutions (ω_i_ = 1), Turkey is distributed the highest water satisfactions by the four asymmetric solutions of ω^NP^, whereas Iraq’s water satisfactions are relatively significantly reduced, showing that allocation solutions are vulnerable to external negotiation power determined by various factors, and Turkey has the leading position in the negotiation over water resources in the ERB.

In addition, disparate DUP vectors result in different allocation solutions by the ANBS. By comparing the three solutions under MTBT-based, MRDC-based, and MENP-based DUPs with ω^NP^ in Figure 5, it can be clearly seen that the MTBT favors Iraq the most since this country is the only country that receives MWR under the MTBT. Moreover, the solution under the MENP-based DUPs seems to be the compromise between the other solutions under the MTBT- and MRDC-based DUPs. Therefore, this solution is relatively unbiased toward any party from the perspective of water satisfaction. At the same time, the MTBT is proposed based on agents’ NPs, resulting in its allocation solution being relatively more realistic. The asymmetric Nash bargaining solution under MENP-based DUPs and ω^NP^ is recommended as the most favorable alternative for the ERB, in the sense that it synthetically considers multidimensional factors relevant to realistic water allocation in the studied basin.

It should be stressed in particular that we do not claim that the suggested allocation framework is a comprehensive method that can consider all relevant criteria and aspects in deriving equitable and reasonable allocation of the shared river resources. For example, it ignores the secondary allocation of the economic value of water resource utilization, for example for hydropower generation, navigation, fisheries, and entertainment, which may lead to economically less efficient water use. Nevertheless, three contributions support the proposed framework as appropriate as an effective tool to facilitate negotiation over water allocations. First, it adopts the DEA-based MCDM approach to synthetically incorporate the agents’ asymmetric information in the social, economic, and political aspects into water allocations within the river basin, and thus more attributes for reasonable reallocation of the shared water resources as stipulated by the Article 6 of the UN Watercourses Convention [7] are taken into account when allocating water resources. Second, it proposes three methods for determining agents’ DUPs, thereby enriching the application of bankruptcy theory and the ANBS in the water resource field. Third, it provides realistic alternative solutions to water allocation in the ERB.

## 4. Conclusions

Transboundary water governance is challenged by multiple local groups with varying asymmetric social, economic, and political status and needs. Effective negotiations among riparian areas over water allocations can prevent hydro-hegemony, but the negotiation process is not necessarily symmetrical, and asymmetry in power will inevitably influence the final negotiation outcome. Therefore, the agents’ bargaining powers should be taken into account in river-sharing problems when available. The ANBS, generalized by the Nash bargaining solution, could offer a good mathematical framework to simulate competition and cooperation among involved agents, and ensure mutually beneficial resolutions for water sharing problems.

This paper developed an asymmetric bargaining model for water allocations in the transboundary river basin, consisting of the following three parts: (1) the DEA is chosen as the MCDM approach to define negotiator power by synthesizing their asymmetric information in social, economic, and political aspects; (2) it develops three methods based on traditional bankruptcy approach and the PRO to generate agent’ DUPs as the starting points of bargaining for water allocations; and (3) it uses the ANBS to establish a water allocation model for the transboundary river by integrating the outcomes from the first two steps and corresponding constraints involving Pareto efficiency, claim boundedness, and non-negativity. The ERB with three littoral states is used to demonstrate how the proposed river allocation model can be helpful in practice.

The numerical results show Turkey receives the highest NP value of 0.4036 since it dominates in terms of economic strength, regional and global political influence, and military capacity, indicating the highest influential capacity in the ERB negotiation. Syria´s NP value is 0.3266. The NP value of Iraq is the lowest at 0.2698, mainly due to its internal political fragmentation and weak military status. The asymmetric Nash solution under the MENP-based DUPs and ω^NP^ is identified as the most favorable alternative for ERB management since it considers more attributes relevant to realistic water allocation in this river basin. The water satisfaction percentages of Turkey, Syria, and Iraq within the ERB under the best alternative identified are 96.30%, 84.23% and 40.88%, respectively. The findings highlight that the acceptability of allocation solutions is sensitive to multidimensional factors and circumstances, and these factors need to be considered simultaneously for creating more realistic negotiations for water allocation. The proposed framework is recommended as an effective tool to facilitate negotiation over practical water allocation within transboundary river basins.

## Figures and Tables

**Table 1:** The contribution and claim (MCM/year) of the three countries in the Euphrates River Basin.

Agent	Composition of Length ^1^	Contribution of Flow ^2^	Water Claim ^3^
Turkey	1230 km (41%)	31,580 (89%)	14,000 (26%)
Syria	710 km (23%)	4000 (11%)	12,600 (23%)
Iraq	1060 km (36%)	0 (0%)	28,100 (51%)
Total	3000 km (100%)	35,580 (100%)	54,700 (100%)

Data sources: ^1^ from Kolar [55]; ^2^ from Lupu [56] and Ibrahim and Sonmez [57]; ^3^ from Mianabadi et al. [13], Mianabadi et al. [34], and Beaumont [54].

**Table 2:** Criteria and indicators applied to estimate the three countries’ negotiation power in the Euphrates River Basin (out of 10).

Agent	Economic Independence and Self Sufficiency				Military Status			International Support			Political Influence and Structure	
	GNI/Capita	Gini Coefficien ^1^	Net Trade/GDP	GDP/Energy Consumption	Yearly Military Expenditures	Military Expenditures/GDP	Armed Forces Personnel/Total Population	U.S. Financial Support ^3^	U.S.Political Support ^4^	Russian Political Support ^4^	Political Influence ^5^	Democracy Level ^6^
Turkey	10.00	10.00	8.87	10.00	10.00	4.79	4.00	0.78	10.00	8.00	10.00	10.00
Syria	2.09	9.04 ^2^	10.00	2.33	1.20	10.00	10.00	0.06	0.00	10.00	7.21	4.14
Iraq	2.57	7.22	9.12	3.17	0.93	4.46	3.70	10.00	2.00	8.00	5.65	7.04

Data source: the World Bank, https://data.worldbank.org/country (2005) [77]. ^1^ Gini coefficient of Turkey and Iraq in 2006. ^2^ Gini coefficient of Syria in 2004. ^3^ U.S. Aid Budget, https://explorer.usaid.gov/cd (2005). ^4^ Depends on the attitudes two superpowers of (United States and Russia). ^5^ The arithmetic average of other indicators [78]. ^6^ The Economist Intelligence Unit, https://www.yabiladi.com/img/content/EIU-Democracy-Index-2015.pdf (2006) [79]. GNI: gross national income.

**Table 3:** Optimal efficiency value and the agents’ negotiation powers (NPs) based on data envelopment analysis (DEA) in the Euphrates River Basin.

Agent	Optimal Efficiency Value	NPs
Turkey	8.7485	0.4036
Syria	7.0802	0.3266
Iraq	5.8474	0.2698
Total	21.6761	1.0000

**Table 4:** Minimum water rights (MWRs) and disagreement utility points (DUPs) for the three countries in the Euphrates River Basin under three methods. MTBT: method for defining agents’ MWRs based on traditional bankruptcy theory. MRDC: method for defining agents’ MWRs based on PRO division with respect to their rates of demand and contribution. MENP: method for defining agents’ MWRs based on PRO division with respect to their external NPs.

Agent	MTBT		MRDC		MENP	
	MWRs	DUPs	MWRs	DUPs	MWRs	DUPs
Turkey	0.00	0.000	4952.78	0.354	3675.35	0.263
Syria	0.00	0.000	2409.76	0.191	2677.03	0.212
Iraq	8980.00	0.320	2962.77	0.105	4930.70	0.175

**Table 5:** Solutions derived from four classical bankruptcy rules of PRO, CEA, CEL, and AP.

Agent	Classical bankruptcy solutions							
	PRO		CEA		CEL		AP	
	xi	pi	xi	pi	xi	pi	xi	pi
Turkey	9106.40	65.05%	11,860.00	84.71%	7626.67	54.48%	8145.23	58.18%
Syria	8195.76	65.05%	11,860.00	94.13%	6226.67	49.42%	7330.71	58.18%
Iraq	18,277.84	65.05%	11,860.00	42.21%	21,726.67	77.32%	20,104.06	71.54%
Total	35,580.00	/	35,580.00	/	35,580.00	/	35,580.00	/

“xi” denotes that the water resource allocated to agent i; “pi” denotes water satisfaction of riparian i.

**Table 6:** Multiple alternative solutions derived from the asymmetric bargaining framework under different NP and DUP vectors.

Agent	DUPs	Nash Bargaining Solutions							
		ω_i_ = 1		ω_i_ = ω^Rc^		ω_i_ = ω^Ra^		ω_i_ = ω^NP^	
		xi	pi	xi	pi	xi	pi	xi	pi
Turkey	0.000	11,860.00	84.71%	9108.48	65.06%	14,000.00	100.00%	14,000.00	100.00%
Syria	0.000	11,860.00	94.13%	8194.07	65.03%	12,600.00	100.00%	11,817.62	93.79%
Iraq	0.000	11,860.00	42.21%	18,277.45	65.04%	8980.00	31.96%	9762.38	34.74%
Turkey	0.000	8866.66	63.33%	6809.59	48.64%	14,000.00	100.00%	10,735.76	76.68%
Syria	0.000	8866.67	70.37%	6125.99	48.62%	12,600.00	100.00%	8687.56	68.95%
Iraq	0.320	17,846.67	63.51%	22,644.42	80.59%	8980.00	31.96%	16,156.68	57.50%
Turkey	0.354	13,371.01	95.51%	11,417.98	81.56%	14,000.00	100.00%	14,000.00	100.00%
Syria	0.191	10,827.99	85.94%	8225.92	65.29%	12,600.00	100.00%	11,285.28	89.57%
Iraq	0.105	11,381.00	40.50%	15,936.10	56.71%	8980.00	31.96%	10,294.72	36.64%
Turkey	0.263	11,774.32	84.10%	9895.36	70.68%	14,000.00	100.00%	13,481.60	96.30%
Syria	0.212	10,776.00	85.52%	8272.61	65.66%	12,600.00	100.00%	10,612.40	84.23%
Iraq	0.175	13,029.67	46.37%	17,412.03	61.96%	8980.00	31.96%	11,486.00	40.88%

d_i_ = 0 indicates that the riparian country’s DUP is 0; ω_i_ = 1 indicates that the riparian countries are given equal weights, i.e., the bargaining weight (BW) vector is (1,1,1); ω_i_ = ω^Rc^ indicates that the BW vector of the riparian countries is determined only by their water demands; ω_i_ = ω^Ra^ indicates that the BW vector of the riparian countries is determined only by their water contributions; and ω_i_ = ω^NP^ indicates that the BW vector of the riparian countries is defined by their external negotiation power.

## Abbreviations

ANBS	Asymmetric Nash bargaining solution
ERB	Euphrates River Basin
CG	Cooperative game
BWs	Bargaining weights
DUPs	Disagreement utility points
MCDM	Multi-criteria decision-making
DEA	Data envelopment analysis
NPs	Negotiation powers
MWRs	Minimum water rights
MCM	Million cubic meters
FAO	Food and Agriculture Organization of the United Nations
GAP	Guneydogu Anadolu Projesi
GDP	Gross domestic product
GNI	Gross national income
DMU	Decision making unit
PRO	Proportional
MTBT	Method for defining agents’ MWRs based on traditional bankruptcy theory
MRDC	Method for defining agents’ MWRs based on PRO division with respect to their rates of demand and contribution
MENP	Method for defining agents’ MWRs based on PRO division with respect to their external NPs
CEA	Constrained equal award
CEL	Constrained equal loss
AP	Adjusted proportional
